# Risk factors for delayed social‐emotional development and behavior problems at age two: Results from the All Our Babies/Families (AOB/F) cohort

**DOI:** 10.1002/hsr2.82

**Published:** 2018-08-28

**Authors:** Sheila W. McDonald, Heather L. Kehler, Suzanne C. Tough

**Affiliations:** ^1^ Departments of Pediatrics and Community Health Sciences, Cumming School of Medicine University of Calgary Calgary Alberta Canada; ^2^ Child Development Centre University of Calgary Calgary Alberta Canada

**Keywords:** behavior problems, child development, cohort study, risk factors, social‐emotional development

## Abstract

**Background and Aims:**

Social‐emotional delays and behavioral problems at preschool age are associated with negative outcomes at school age, including ongoing behavior problems, poorer social functioning, and academic difficulties. Understanding modifiable risk factors for suboptimal development requires consideration of contemporary family circumstances to determine areas for effective early intervention to optimize development. This study aimed to identify risk factors for delayed social‐emotional development and behavior problems at age two among participants of the All Our Babies/Families cohort study.

**Methods:**

Mothers (*N* = 1596) completed five comprehensive questionnaires spanning midpregnancy to 2 years postpartum. At child age two, behavior and competence outcomes were measured using the Brief Infant‐Toddler Social and Emotional Assessment. Chi square analysis and multivariable logistic regression modeling was used to identify key risk factors for suboptimal child outcomes. Predicted probabilities for adverse outcomes in the presence of risk were calculated.

**Results:**

Risk factors for possible delayed social‐emotional development in children included maternal depression at 2 years postpartum (OR 2.46, 95% CI 1.63, 3.72), lower parenting self‐efficacy at 2 years postpartum (OR 2.76, 95% CI 1.51, 5.06), non‐daily play‐based interaction when child was 1 and 2 years old (OR 1.43, 95% CI 1.02, 1.99), child delayed sleep initiation at 2 years of age (OR 1.58, 95% CI 1.05, 2.37), and playgroup non‐attendance between 1 and 2 years postpartum (OR 1.43, 95% CI 1.03, 1.99). Risk factors for possible behavior problems included lower maternal optimism during pregnancy (OR 2.02, 95% CI 1.36, 2.99), maternal depression at 2 years postpartum (OR 2.19, 95% CI 1.46, 3.27), difficulty balancing responsibilities at 2 years postpartum (OR 2.32 95% CI 1.55, 3.47), child second language exposure at 2 years of age (OR 1.88, 95% CI 1.37, 2.58), child delayed sleep initiation at 2 years of age (OR 1.55 95% CI 1.06, 2.26), child frequent night wakings at 2 years of age (OR 2.95 95% CI 2.13, 4.10), and more screentime exposure at 2 years of age (OR 1.85 95% CI 1.34, 2.54).

**Conclusions:**

This study suggests that addressing maternal mental health and promoting parenting strategies that encourage play‐based interaction, limiting screen time, preventing sleep problems, and engagement in informal playgroups would reduce the risk of behavior and social‐emotional problems.

## 1 INTRODUCTION

Social‐emotional and behavioral problems affect an estimated 5% to 20% of preschool‐aged children (<5 years) across a number of diverse populations, contexts, and assessment methods.^1^, ^2^, ^3^ In Canada, population profiles using the Early Development Index, an aggregate based indicator, found that over 40% of children in kindergarten (age 5 years) were vulnerable in social‐emotional health.^4^ Social‐emotional competencies in young children include helping, sharing, comforting, and exhibiting empathy and compliance, while behavioral problems include disruptive behavior, inhibition of impulse control, aggression, defiance, over‐activity, negative emotionality, anxiety, and withdrawal.^5^ Social‐emotional delays and behavioral problems at preschool age are associated with increased risk for negative outcomes at school age, including ongoing behavior problems, psychiatric disorders, and poorer academic achievement.^6^, ^7^ The development of social‐emotional competencies has important consequences for young children, including the ability to make and maintain quality friendships, and peer acceptance.^8^ Young children with social‐emotional competencies are more likely to experience positive early school adjustment and academic success.^9^

Early identification of children with delayed social‐emotional competencies and behavioral problems provides an opportunity for parents and care providers to implement strategies to remediate problems before school entry.^10^ Understanding the factors that contribute to the development of poor social‐emotional competencies and behavioral problems in the first few years of life will help mitigate long‐term adverse consequences and trajectories associated with suboptimal behavior. From a policy, program, and return on investment perspective, early interventions are cost‐effective and have greater positive impact than later remediation.^11^, ^12^

Despite evidence about the importance of early identification of children with delayed social‐emotional development and behavior problems,^13^, ^14^ there is limited Canadian research about family and community factors that may prevent poor outcomes within the context of pregnancy, and the early postpartum period. Assessing a breadth of risk factors helps inform independent effects and potential targets for early intervention. In addition, contemporary information is necessary to determine the magnitude of influence these factors have, within the current social context of dual parent working families, child care, screen time, and time stresses.

The objective of this study was to identify key risk factors for delayed social‐emotional development and behavioral problems among 2‐year‐old children in a community sample of mothers and their children. Data were analyzed from the All Our Babies/Families (AOB/F) cohort study,^15^ which contains prospectively collected information on maternal, family, child care, parenting, and community factors related to development. This population‐based cohort of women, recruited in Calgary, Canada, is demographically similar to the pregnant and parenting populations of westernized urban centers.^15^ These findings will inform and focus policy and programming to improve social‐emotional and behavior development in the preschool years.

## 2 METHODS

### 2.1 Study design

The AOB/F study is an ongoing prospective population‐based pregnancy cohort study in Calgary, Canada, that began in 2008.^15^ The AOB/F study was developed to investigate the relationships between the prenatal and early life period, and outcomes for infants, children, and mothers. Detailed descriptions of the AOB/F study design and methods have previously been described.^15^ Briefly, the cohort was established using a community‐based recruitment strategy, involving primary health care offices and a citywide laboratory service (Calgary Laboratory Service). Community posters were implemented to obtain a socioeconomically and ethnically diverse sample of women representative of the parenting population in an urban Canadian center. Recruitment began in May 2008 and was completed in December 2010. Women were eligible to participate if they were less than 25‐week gestation, 18 years of age or older, resided in the greater Calgary area, and were able to complete the written questionnaires in English. Prior to inclusion in the study, all participants provided informed consent. Participants were asked to complete three written questionnaires: (1) at study intake (second trimester), (2) during their third trimester, and (3) at 4 months postpartum. Information was collected on pregnancy history, demographics, lifestyle, health care utilization, physical and mental health, birth outcomes, child health, and parenting. Participants were also asked to consent to the research team accessing their obstetrical and birth records. In total, 3317 women consented to participate and completed at least one questionnaire. Women who consented to be contacted for future research were asked to participate in subsequent follow‐up questionnaires when their child was 1 and 2 years old. The design of the 2‐year follow‐up questionnaire was delayed due to logistical constraints including receipt of funding and ethical approval; as such, not all original study participants were eligible at 2 years, since some children were out of the relevant age range. Among those eligible (*n* = 2114), 1596 completed and returned the 2‐year questionnaire, for a participation rate of 75.5%. From the original total sample of participants (*n* = 3317), this corresponded to 48.1% participating in the 2‐year questionnaire.

At 2 years, the questionnaire collected information about their child's health, child care environment, child development, health service utilization, community resource use, demographics, maternal lifestyle, maternal mental health, work status, and parenting experience. All questionnaires were developed with input from health care providers, community program experts, and researchers with expertise in child development and family well‐being. Standardized measures were included as part of the questionnaires when available. Ethical approval for the study was provided by the Conjoint Health Research Ethics Board of the Faculty of Medicine at the University of Calgary.

### 2.2 Main outcomes

The Brief Infant‐Toddler Social and Emotional Assessment (BITSEA) is a concise screening instrument to identify possible behavior problems and delayed social‐emotional competencies in children aged 12 to 36 months.^16^ It was designed to be completed by parents or caregivers and consists of 42 items. The BITSEA measures two domains, behavior problems (eg, aggression, defiance, over‐activity, negative emotionality, anxiety, and withdrawal) and delayed social‐emotional competencies (eg, empathy, pro‐social behaviors, and compliance).^16^ A total behavioral problem score and a total social‐emotional competencies score are derived by summing relevant items for each domain subscale. Using the BITSEA standardized scoring cut‐offs from the published normative data, children were categorized with possible behavioral problems if they scored at or above the 75^th^ percentile of normative data, and children were categorized with possible delayed social‐emotional competencies if they scored at or below the 15^th^ percentile of normative data.^16^ The BITSEA has shown excellent test‐retest reliability (behavior problems scale: *r* = 0.87; social‐emotional competence scale: *r* = 0.85) and good interrater agreement between mothers and fathers (behavior problems scale: *r* = 0.68; social‐emotional competence scale: *r* = 0.61).^17^ Internal consistency measured by Cronbach's alpha was 0.79 for the behavior problems scale and 0.65 for the social‐emotional competence scale.^17^ Similarly, in the current sample, internal consistency was 0.75 for the behavior problem scale and 0.63 for the social‐emotional competence scale. Given the large size of our sample, we used a parent report screening measure to identify children with symptoms of behavioral problems and social emotional difficulties. Screening tools are often used in population‐based studies due to their feasibility and ease of administration compared with more time‐consuming and costly diagnostic interviews. Of note, the use of delayed social‐emotional development and behavior problems throughout the manuscript refer to “possible” delays and problems.

### 2.3 Independent variables

Information on a broad range of potential risk factors collected during pregnancy and the first 2 years postpartum were examined in the current study and were grouped into four broad categories: (1) socio‐demographic and adverse life event factors, (2) pregnancy and birth outcome factors, (3) 1‐year postpartum factors, and (4) 2‐year postpartum factors. The socio‐demographic factors examined were maternal age, marital status, maternal education, and household income. Adverse life event factors included a history of a mental health disorder (s) and a history of experiencing abuse (physical, emotional, sexual, financial, or neglect). Factors measured during pregnancy included maternal depressive symptoms measured by the Edinburgh Postnatal Depression Scale (EPDS),^18^ maternal anxiety symptoms measured by the Spielberger State Anxiety Inventory (SSAI),^19^ maternal stress measured by the Perceived Stress Scale (PSS),^20^ maternal social support measured by the MOS Social Support Scale (MOS‐SSS),^21^ and maternal optimism measured by the Life‐Orientation Test‐Revised (LOT‐R).^22^ Birth outcome factors included gestational age at birth, birth weight, and child sex. One‐year postpartum factors included maternal depressive symptoms measured by the EPDS,^18^ maternal anxiety symptoms measured by the SSAI,^19^ maternal stress measured by the PSS,^20^ maternal social support measured by the National Longitudinal Survey of Children and Youth (NLSCY) Social Support Scale,^23^ maternal relationship happiness, and co‐parenting agreement. Two‐year postpartum maternal factors included depressive symptoms measured by the Centre for Epidemiologic Studies Depression Scale (CES‐D),^24^ anxiety symptoms measured by the SSAI,^19^ social support measured by the NLSCY Social Support Scale,^23^ parenting self‐efficacy measured by the Parenting Sense of Competence Scale (PSOC),^25^ work‐life balance, parent‐child interaction (reading and playing), and community resource use (formal parenting program/group, informal playgroup). Two‐year‐old child factors included child care arrangement, second language exposure, sleep habits (sleep initiation, napping, night waking), and screen time. For the purposes of modeling, all factors were dichotomized into a possible risk and reference category. Validated cut‐offs were used for the EPDS, SSAI, and MOS‐SSS. For those scales without a validated cut‐off, including the PSS, NLSCY Social Support Scale, PSOC, and LOT‐R, one standard deviation above or below the mean, depending on scoring, was used to define “at‐risk” status. For all standardized scales, the specific cut‐off values used have been noted in Tables 2 and 4.

### 2.4 Data analysis

Descriptive statistics were used to describe participants socio‐demographics, maternal mental health at 2 years postpartum, and child social‐emotional and behavioral functioning at 2 years. Among those eligible for the 2‐year questionnaire (*n* = 2114), socio‐demographic characteristics, birth outcomes, and maternal mental health symptoms during pregnancy and at 1 year postpartum were compared between participants and non‐participants of the 2‐year questionnaire using Pearson's Chi‐square Test. An initial bivariate analysis using Pearson's Chi‐square test was conducted to identify risk factors for delayed social‐emotional development and child behavioral problems at age two. Risk factors identified at the bivariate level based on statistical significance at the *P* < 0.10 level were considered for inclusion in the subsequent multivariable logistic regression models. A manual stepwise approach was used to build the multivariable models, with blocks of variables being added in sequential steps in the following order: (1) socio‐demographic and adverse life event variables, (2) pregnancy and birth outcome variables, (3) 1‐year postpartum variables, and (4) 2‐year postpartum variables. To best address the research objectives, these blocks were ordered conceptually in temporal order and from least to most modifiable, as per our model building strategy used in previous research in the AOF.^26^, ^27^, ^28^ Risk factors within each block were identified for inclusion based on the literature. Statistical significance was set at *P* < 0.05 for inclusion in the next step of the logistic regression modeling, and parsimonious models were developed at each block. As a final step, we entered each factor that was dropped at previous steps to ensure model robustness. Multivariable logistic regression results are presented as odds ratios (ORs) and 95% confidence intervals (CIs). Predicted probabilities for delayed social‐emotional development and behavior problems were calculated for the combination of risk factors identified from the final models. Stata Version 12.1 was used for the statistical analysis.

## 3 RESULTS

### 3.1 Maternal participant characteristics

The characteristics of mothers who participated in the 2‐year questionnaire (*n* = 1596) are described in Table 1. Compared with women who were eligible for the 2‐year questionnaire but did not participate, women who participated in the 2‐year questionnaire were more likely to have post‐secondary education and household incomes greater than $80 000 CAD and be married or in a common law relationship and Caucasian (all *P* < 0.05). Two‐year questionnaire participants were less likely to both experience symptoms of anxiety during pregnancy and have a preterm birth, than non‐participants (all *P* < 0.05). There were no differences in maternal age, maternal country of birth, pre‐pregnancy mental health history, depressive symptoms during pregnancy, child birthweight, and mental health symptoms at 1‐year postpartum between participants and non‐participants.

**Table 1:** Participant characteristics

Characteristic	*N* = 1596 *N* (%)
Maternal age at delivery	
24 or younger	78 (4.89)
25‐34 years of age	1085 (67.98)
35 or older	360 (22.56)
Missing	73 (4.57)
Marital status at 2 years postpartum	
Married/common law	1536 (96.24)
Single/separated/divorced/widowed	60 (3.76)
Missing	0 (0.00)
Maternal education at study intake	
Has not completed post‐secondary	347 (21.74)
Graduated post‐secondary	1237 (77.51)
Missing	12 (0.75)
Household income before taxes and deductions at study intake	
Less than $60 000	207 (12.97)
$60 000‐$79 999	211 (13.22)
$80 000 or more	1107 (69.36)
Missing	71 (4.45)
Mother was born in Canada	
Yes	1276 (79.95)
No	309 (19.36)
Missing	11 (0.69)
Maternal race	
White/Caucasian	1301 (81.52)
Other	283 (17.73)
Missing	12 (0.75)
Maternal depressive symptoms at 2 years postpartum (CES‐D)	
No (CES‐D < 16)	1396 (87.47)
Yes (CES‐D ≥ 16)	200 (12.53)
Missing	0 (0.00)
Maternal anxiety symptoms at 2 years postpartum (SSAI)	
No (SSAI <40)	1334 (83.58)
Yes (SSAI ≥40)	242 (15.16)
Missing	20 (1.25)
Child social‐emotional competencies at age 2 years (BITSEA)	
No delay	1369 (85.78)
Possible delay	210 (13.16)
Missing	17 (1.07)
Child behavior problems at age 2 years (BITSEA)	
No behavior problems	1344 (84.21)
Possible behavior problems	236 (14.79)
Missing	16 (1.00)

Abbreviations: BITSEA, Brief Infant‐Toddler Social and Emotional Assessment; CES‐D, Centre for Epidemiologic Studies Depression Scale; SSAI, Spielberger State Anxiety Inventory.

### 3.2 Child development outcomes at 2 years

At questionnaire completion, children were an average age of 2.03 years (sd = 0.10, range = 1.88‐2.84 years). Based on maternal report on the BITSEA, 13% (*n* = 210/1579) of children were identified with possible delayed social‐emotional development and 15% (*n* = 236/1580) of children with possible behavior problems at age two (Table 1).

### 3.3 Risk factors for delayed social‐emotional development at age two

Bivariate associations between all potential risk factors examined and delayed social‐emotional development at age two are summarized in Table 2. Multivariable modelling identified the combination of risk factors most predictive of delayed social‐emotional development at age two, which included maternal depression at 2 years postpartum (OR 2.46, 95% CI 1.63, 3.72), lower parenting self‐efficacy at 2 years postpartum (OR 2.76, 95% CI 1.51, 5.06), lack of parent‐child engagement in daily imitation play at 1 and 2 years (OR 1.43, 95% CI 1.02, 1.99), more than 30 minutes to initiate child night sleep at 2 years of age (OR 1.58, 95% CI 1.05, 2.37), and not attending an informal play group between 1 and 2 years postpartum (OR 1.43, 95% CI 1.03, 1.99) (Table 3). The predicted probability of delayed social‐emotional development at age 2 was 65% in the presence of all five risk factors.

**Table 2:** Risk factors for delayed social‐emotional development at age 2

Category	Risk Factor	No Delay (BITSEA) *N* = 1369 *N* (%)	Possible Delay (BITSEA) *N* = 210 *N* (%)	Pearson Chi‐Square Test X^2^, *P*‐value
Socio‐demographic and adverse life event factors	Maternal age at delivery			
	34 years old or less	1018 (77.18)	142 (70.65)	4.12, 0.042
	35 years old or more	301 (22.82)	59 (29.35)	
	Marital status at 2 years postpartum			0.11, 0.741
	Married or common law	1317 (96.20)	203 (96.67)	
	Single, separated, divorced, widowed	52 (3.80)	7 (3.33)	
	Maternal education at study intake			0.63, 0.426
	Has not completed post‐secondary	294 (21.59)	50 (24.04)	
	Graduated post‐secondary	1068 (78.41)	158 (75.96)	
	Household income at 1 year postpartum			4.42, 0.035
	Less than $80 000	339 (30.19)	66 (38.15)	
	$80 000 or more	784 (69.81)	107 (61.85)	
	History of any mental health disorder			1.62, 0.204
	Yes	441 (32.40)	77 (36.84)	
	No	920 (67.60)	132 (63.16)	
	History of ever experiencing abuse (physical, emotional, sexual, financial, neglect)			0.80, 0.370
	Yes	325 (24.38)	45 (21.53)	
	No	1008 (75.62)	164 (78.47)	
Pregnancy and birth outcome factors	Depressive symptoms during pregnancy (EPDS)			5.21, 0.022
	Yes (EPDS ≥13)	85 (6.25)	22 (10.53)	
	No (EPDS <13)	1275 (93.75)	187 (89.47)	
	Anxiety symptoms during pregnancy (SSAI)			4.66, 0.031
	Yes (SSAI ≥40)	174 (13.03)	38 (18.63)	
	No (SSAI <40)	1161 (86.97)	166 (81.37)	
	Stress during pregnancy (PSS) Higher (PSS ≥20)			8.15, 0.004
	Lower (PSS <20)	184 (13.57)	44 (21.05)	
		1172 (86.43)	165 (78.95)	
	Social support during pregnancy (MOS)			20.96, <0.001
	Higher (MOS >69)	1214 (89.79)	164 (78.85)	
	Lower (MOS ≤69)	138 (10.21)	44 (21.15)	
	Optimism during pregnancy (LOT‐R)			6.10, 0.013
	Higher (LOT‐*R* > 13)	1171 (87.85)	169 (81.64)	
	Lower (LOT‐R ≤ 13)	162 (12.15)	38 (18.36)	
	Gestational age at delivery			2.70, 0.100
	Preterm (<37 weeks)	85 (6.37)	19 (9.50)	
	Term (≥37 weeks)	1250 (93.63)	181 (90.50)	
	Birth weight			3.61, 0.057
	Low birth weight (<2500 grams)	69 (5.43) 1202 (94.57)	17 (8.90) 174 (91.10)	
	Not low birth weight (≥2500 grams)			
	Child sex			3.67, 0.055
	Male	685 (50.97)	117 (58.21)	
	Female	659 (49.03)	84 (41.79)	
1 year postpartum factors	Depressive symptoms (EPDS)			11.07, 0.001
	Yes (EPDS ≥13)			
	No (EPDS <13)	57 (5.05) 1071 (94.95)	20 (11.43) 155 (88.57)	
	Anxiety symptoms (SSAI)			9.73, 0.002
	Yes (SSAI ≥40)	166 (15.09)	41 (24.70)	
	No (SSAI <40)	934 (84.91)	125 (75.30)	
	Stress symptoms (PSS)			9.14, 0.002
	Higher (PSS ≥19)	210 (18.95)	49 (28.99)	
	Lower (PSS <19)	898 (81.05)	120 (71.01)	
	Social support (NLSCY)			2.83, 0.093
	Higher (NLSCY >17)	921 (81.50)	134 (76.14)	
	Lower (NLSCY ≤17)	209 (18.50)	42 (23.86)	
	Relationship happiness			10.30, 0.001
	Happy	1032 (92.39)	148 (85.06)	
	Unhappy	85 (7.61)	26 (14.94)	
	Co‐parenting agreement			6.25, 0.012
	Agree	961 (84.74)	136 (77.27)	
	Not sure or disagree	173 (15.26)	40 (22.73)	
2‐year postpartum factors	Depressive symptoms (CES‐D)			30.47, <0.001
	Yes (CES‐D ≥ 16)	147 (10.74)	51 (24.29)	
	No (CES‐D < 16)	1222 (89.26)	159 (75.71)	
	Anxiety symptoms (SSAI)			22.10, <0.001
	Yes (SSAI ≥40)	185 (13.70)	55 (26.32)	
	No (SSAI <40)	1165 (86.30)	154 (73.68)	
	Social support (NLSCY)			11.25, 0.001
	Higher (NLSCY >17)	1185 (86.81)	164 (78.10)	
	Lower (NLSCY ≤17)	180 (13.19)	46 (21.90)	
	Parenting self‐efficacy (PSOC)			32.78, <0.001
	Higher (PSOC >17) Lower (PSOC ≤17)	1322 (96.78)	185 (88.10)	
		44 (3.22)	25 (11.90)	
	Child was consistently read to daily at 1 and 2 years			9.59, 0.002
	Yes	832 (74.09)	110 (62.86)	
	No	291 (25.91)	65 (37.14)	
	An adult in the household consistently played imitation games with the child daily at 1 and 2 years			9.18, 0.002
	Yes	743 (65.75)	95 (53.98)	
	No	387 (34.25)	81 (46.02)	
	Mother's perceived ability to fulfill family, work, or other responsibilities			2.67, 0.102
	It is never or sometimes difficult	1214 (88.68) 155 (11.32)	178 (84.76) 32 (15.24)	
	It is difficult most of the time/it is always difficult			
	Mother attended a parenting group, program, or center in the past year			0.20, 0.659
	Yes	384 (28.05)	62 (29.52)	
	No	985 (71.95)	148 (70.48)	
	Mother attended an informal play group in the past year			9.29, 0.002
	Yes	830 (60.63)	104 (49.52)	
	No	539 (39.37)	106 (50.48)	
	Childcare arrangement at 2 years			0.92, 0.337
	Parent/relative/nanny	856 (62.76)	139 (66.19)	
	Childcare center/day home	508 (37.24)	71 (33.81)	
	Child regular second language exposure at 2 years			5.08, 0.024
	Yes	379 (27.68)	74 (35.24)	
	No	990 (72.32)	136 (64.76)	
	Child night sleep initiation time at 2 years (in general)			4.87, 0.027
	30 min or less	1175 (85.83)	168 (80.00)	
	More than 30 min	194 (14.17)	42 (20.00)	
	Total time child spends napping per day at 2 years (average)			0.36, 0.548
	Less than 1 h	71 (5.19)	13 (6.19)	
	At least 1 h	1297 (94.81)	197 (93.81)	
	How often child wakes at night at 2 years (in general)			0.04, 0.841
	2 or fewer times per week	1071 (78.23)	163 (77.62)	
	3 or more times per week	298 (21.77)	47 (22.38)	
	Child total screen time per day at 2 years (television, movies, computer/tablet)			2.46, 0.117
	Less than 1 h	686 (51.73)	94 (45.85)	
	1 h or more	640 (48.27)	111 (54.15)	

Abbreviations: BITSEA, Brief Infant Toddler Social Emotional Assessment; CES‐D, Centre for Epidemiologic Studies Depression Scale; EPDS, Edinburgh Postnatal Depression Scale; LOT‐R, Life‐Orientation Test‐Revised; MOS, MOS Social Support Scale; NLSCY, National Longitudinal Survey of Children and Youth Social Support Scale; PSOC, Parenting Sense of Competence Scale; PSS, Perceived Stress Scale; SSAI, Spielberger State Anxiety Inventory.

**Table 3:** Multivariable model of risk factors for delayed social‐emotional development at age 2

Risk Factor	Adjusted Odds Ratio	95% Confidence Interval
Maternal depressive symptoms at 2 years postpartum (CES‐D) Yes (CES‐D ≥ 16) No (CES‐D < 16)	2.46 Reference	[1.63, 3.72]*
Parenting self‐efficacy at 2 years postpartum (PSOC) Lower (PSOC ≤17.98) Higher (PSOC >17.98)	2.76 Reference	[1.51, 5.06]*
Consistent daily play with an adult at 1 and 2 years No Yes	1.43 Reference	[1.02, 1.99]*
Child night sleep initiation time at 2 years (in general) More than 30 min 30 min or less	1.58 Reference	[1.05, 2.37]*
Mother attended an informal play group when child was between the ages of 1 and 2 No Yes	1.43 Reference	[1.03, 1.99]*

Abbreviations: CES‐D, Centre for Epidemiologic Studies Depression Scale; PSOC, Parenting Sense of Competence Scale.

* *P* < 0.05.

### 3.4 Risk factors for behavior problems at age two

Bivariate associations between all potential risk factors examined and child behavior problems at age two are summarized in Table 4. Multivariable modeling revealed the combination of risk factors most predictive of behavior problems at age two to be having a mother who reported lower optimism during pregnancy (OR 2.02, 95% CI 1.36, 2.99), having a mother experiencing depression at 2 years postpartum (OR 2.19, 95% CI 1.46, 3.27), having a mother who reported more difficulty balancing family, work, and other responsibilities at 2 years postpartum (OR 2.32 95% CI 1.55, 3.47), child exposure to a second language on a regular basis at 2 years of age (OR 1.88, 95% CI 1.37, 2.58), more than 30 minutes to initiate child night sleep at 2 years of age (OR 1.55 95% CI 1.06, 2.26), child night wakings at least three times a week at 2 years of age (OR 2.95 95% CI 2.13, 4.10), and child exposure to at least 1 hour of daily screen time on any type of media (television, movies, computer, tablet, etc.) at 2 years of age (OR 1.85 95% CI 1.34, 2.54) (Table 5). The predicted probability of behavior problems at age 2 was 88% in the presence of all seven risk factors.

**Table 4:** Risk factors for behavioral problems at age 2

Category	Risk Factor	No Problems (BITSEA) *N* = 1344*N* (%)	Possible Behavior Problems (BITSEA)*N* = 236*N* (%)	Pearson Chi‐Square TestX^2^, *P*‐value
Socio‐ demographic and adverse life event factors	Maternal age at delivery 34 years old or less 35 years old or more	982 (75.65) 316 (24.35)	179 (80.27) 44 (19.73)	2.24, 0.134
	Marital status at 2 years postpartum Married or common law Single, separated, divorced, widowed	1299 (96.65) 45 (3.35)	222 (94.07) 14 (5.93)	3.73, 0.053
	Maternal education at study intake Has not completed post‐secondary Graduated post‐secondary	278 (20.78) 1060 (79.22)	67 (28.76) 166 (71.24)	7.37, 0.007
	Household income at 1 year postpartum Less than $80 000 $80 000 or more	324 (29.01) 793 (70.99)	81 (45.25) 98 (54.75)	18.95, <0.001
	History of any mental health disorder Yes No	410 (30.67) 927 (69.33)	108 (46.15) 126 (53.85)	21.62, <0.001
	History of ever experiencing abuse (physical, emotional, sexual, financial, neglect) Yes No	300 (22.83) 1014 (77.17)	71 (31.00) 158 (69.00)	7.13, 0.008
Pregnancy and birth outcome factors	Depressive symptoms during pregnancy (EPDS) Yes (EPDS ≥13) No (EPDS <13)	74 (5.54) 1262 (94.46)	33 (14.10) 201 (85.90)	22.99, <0.001
	Anxiety symptoms during pregnancy (SSAI) Yes (SSAI ≥40) No (SSAI <40)	160 (12.21) 1150 (87.79)	52 (22.61) 178 (77.39)	17.80, <0.001
	Stress during pregnancy (PSS) Higher (PSS ≥20) Lower (PSS <20)	166 (12.46) 1166 (87.54)	62 (26.50) 172 (73.50)	31.51, <0.001
	Social support during pregnancy (MOS) Higher (MOS >69) Lower (MOS ≤69)	1196 (89.86) 135 (10.14)	183 (79.57) 47 (20.43)	20.17, <0.001
	Optimism during pregnancy (LOT‐R) Higher (LOT‐R > 13) Lower (LOT‐R ≤ 13)	1173 (89.20) 142 (10.80)	167 (73.89) 59 (26.11)	39.84, <0.001
	Gestational age at delivery Preterm (<37 weeks) Term (≥37 weeks)	84 (6.40) 1229 (93.60)	20 (8.97) 203 (91.03)	2.00, 0.158
	Birth weight Low birth weight (<2500 grams) Not low birth weight (≥2500 grams)	68 (5.47) 1176 (94.53)	18 (8.22) 201 (91.78)	2.55, 0.110
	Child sex Male Female	691 (52.47) 626 (47.53)	112 (48.91) 117 (51.09)	0.99, 0.320
1 year postpartum factors	Depressive symptoms (EPDS) Yes (EPDS ≥13) No (EPDS <13)	55 (4.91) 1066 (95.09)	22 (12.02) 161 (87.98)	14.34, <0.001
	Anxiety symptoms (SSAI) Yes (SSAI ≥40) No (SSAI <40)	156 (14.21) 942 (85.79)	51 (30.18) 118 (69.82)	27.33, <0.001
	Stress symptoms (PSS) Higher (PSS ≥19) Lower (PSS <19)	197 (17.89) 904 (82.11)	62 (35.03) 115 (64.97)	27.71, <0.001
	Social support (NLSCY) Higher (NLSCY >17) Lower (NLSCY ≤17)	929 (82.65) 195 (17.35)	127 (69.40) 56 (30.60)	17.81, <0.001
	Relationship happiness Happy Unhappy	1029 (92.29) 86 (7.71)	152 (85.88) 25 (14.12)	8.00, 0.005
	Co‐parenting agreement Agree Not sure or disagree	957 (84.84) 171 (15.16)	141 (77.05) 42 (22.95)	7.02, 0.008
2‐year postpartum factors	Depressive symptoms (CES‐D) Yes (CES‐D ≥ 16) No (CES‐D < 16)	134 (9.97) 1210 (90.03)	64 (27.12) 172 (72.88)	53.86, <0.001
	Anxiety symptoms (SSAI) Yes (SSAI ≥40) No (SSAI <40)	179 (13.47) 1150 (86.53)	61 (26.41) 170 (73.59)	25.31, <0.001
	Social support (NLSCY) Higher (NLSCY >17) Lower (NLSCY ≤17)	1175 (87.69) 165 (12.31)	175 (74.15) 61 (25.85)	29.92, <0.001
	Parenting self‐efficacy (PSOC) Higher (PSOC >17) Lower (PSOC ≤17)	1291 (96.20) 51 (3.80)	217 (92.34) 18 (7.66)	7.12, 0.008
	Child was consistently read to daily at 1 and 2 years Yes No	822 (73.52) 296 (26.48)	120 (66.30) 61 (33.70)	4.08, 0.043
	An adult in the household consistently played imitation games with the child daily at 1 and 2 years Yes No	731 (64.98) 394 (35.02)	107 (58.79) 75 (41.21)	2.61, 0.106
	Mother's perceived ability to fulfill family, work, or other responsibilities It is never or sometimes difficult It is difficult most of the time/it is always difficult	1212 (90.18) 132 (9.82)	181 (76.69) 55 (23.31)	34.98, <0.001
	Mother attended a parenting group, program, or center in the past year Yes No	370 (27.53) 974 (72.47)	76 (32.20) 160 (67.80)	2.16, 0.141
	Mother attended an informal play group in the past year Yes No	814 (60.57) 530 (39.43)	120 (50.85) 116 (49.15)	7.84, 0.005
	Childcare arrangement at 2 years Parent/relative/nanny Childcare center/day home	855 (63.81) 485 (36.19)	140 (59.57) 95 (40.43)	1.54, 0.215
	Child regular second language exposure at 2 years Yes No	350 (26.04) 994 (73.96)	104 (44.07) 132 (55.93)	31.86, <0.001
	Child night sleep initiation time at 2 years (in general) 30 min or less More than 30 min	1169 (86.98) 175 (13.02)	175 (74.15) 61 (25.85)	25.99, <0.001
	Total time child spends napping per day at 2 years (average) Less than 1 h At least 1 h	66 (4.91) 1278 (95.09)	18 (7.66) 217 (92.34)	3.00, 0.083
	How often child wakes at night at 2 years (in general) 2 or fewer times per week 3 or more times per week	1094 (81.40) 250 (18.60)	140 (59.32) 96 (40.68)	57.21, <0.001
	Child total screen time per day at 2 years (television, movies, computer/tablet) Less than 1 h 1 h or more	700 (53.68) 604 (46.32)	80 (35.24) 147 (64.76)	26.30, <0.001

Abbreviations: CES‐D, Centre for Epidemiologic Studies Depression Scale; EPDS, Edinburgh Postnatal Depression Scale; LOT‐R, Life‐Orientation Test‐Revised; MOS, MOS Social Support Scale; NLSCY, National Longitudinal Survey of Children and Youth Social Support Scale; PSOC, Parenting Sense of Competence Scale; PSS, Perceived Stress Scale; SSAI, Spielberger State Anxiety Inventory.

**Table 5:** Multivariable model of risk factors for behavioral problems at age 2

Risk Factor	Adjusted Odds Ratio	95% Confidence Interval
Higher optimism during late pregnancy (LOT‐R)		
Lower optimism (LOT‐*R* < 15)	2.02	[1.36, 2.99]*
Higher optimism (LOT‐*R* ≥ 15)	Reference	
Maternal depressive symptoms at 2 years postpartum (CES‐D)		
Yes (CES‐D ≥ 16)		[1.46, 3.27]*
No (CES‐D < 16)	2.19 Reference	
Perceived difficulty fulfilling family, work, or other responsibilities when child was 2 years old		[1.55, 3.47]*
More difficulty	2.32	
Less difficulty	Reference	
Child is regularly exposed to a language other than English at age 2		[1.37, 2.58]*
Yes	1.88	
No	Reference	
Child night sleep initiation time at 2 years (in general)		[1.06, 2.26]*
More than 30 min	1.55	
30 min or less	Reference	
How often child wakes at night at age 2		
3 or more times per week 2 or fewer times per week	2.95 Reference	[2.13, 4.10]*
	
Child screen time per day on any type of media (television, movies, computer/tablet) at age 2		[1.34, 2.54]*
1 h or more	1.85	
Less than 1 h	Reference	

Abbreviations: CES‐D, Centre for Epidemiologic Studies Depression Scale; LOT‐R, Life‐Orientation Test‐Revised.

* *P* < 0.05.

## 4 DISCUSSION

Early behavior and social‐emotional development is important for children's ongoing behavior, social competence, mental health, and academic success. Utilizing data from the AOB/F cohort, we identified factors that were associated with delayed social‐emotional development and behavior problems at age two, providing insight into beneficial targets for interventions.

Women who were experiencing depression when their child was 2 years old were more likely to have a child with delayed social‐emotional competencies and behavior problems than women who were not experiencing depression at 2 years postpartum, controlling for other factors. Our findings align with a recent meta‐analysis of 193 studies examining the association between maternal depression and child behavioral and emotional functioning, which concluded that maternal depression is significantly related to lower levels of positive affect and behavior in young children.^29^ While depression at all time points measured (during pregnancy, four months postpartum, 1 year postpartum, and 2 years postpartum) were each significant in the bivariate analysis for both child development outcomes in the present study, only depression at 2 years postpartum remained in the final multivariable models, which suggests that current maternal mental health status is closely linked to children's current social‐emotional and behavioral development. Consequently, identifying and intervening to support mothers with symptoms of depression could positively impact child social‐emotional and behavioral development.

Having a mother who reported lower parenting self‐efficacy when their child was 2 years old increased the likelihood that their child would have delayed social‐emotional competencies at age two. Perceived parental efficacy describes “beliefs or judgments a parent holds of their capabilities to organize and execute a set of tasks related to parenting a child.”^30^ Our findings align with previous research reporting a positive association between parenting self‐efficacy and children's social competence.^5^ Identifying parents of young children with low parenting self‐efficacy and providing appropriate support and services to enhance parenting self‐efficacy may positively influence children's social‐emotional development.

Children who were not engaged in daily play‐based interactions with their parents at 1 and 2 years were significantly more likely to experience delays in their social‐emotional development at 2 years of age compared with children who were engaged in daily play‐based interactions with their parents. Play contributes to the cognitive, physical, social, and emotional well‐being of children.^31^ In our cohort, over a third of children were not being engaged in consistent daily play‐based interactions with their parents. Encouraging parents or other caregivers to protect time for daily play and encouraging new parents to engage in imitation play would support social emotional development.

Children with sleep onset delays of 30 minutes or greater were more likely to have delayed social‐emotional abilities and behavior problems compared with children with shorter sleep onset. Similarly, behavior problems were more likely for children who experienced frequent night wakings compared with children with less frequent night wakings. Child sleep problems have previously been linked to child behavior problem, poorer learning capacity, and academic performance.^32^ This research builds on these findings, adding that poor sleep habits and difficulty initiating sleep, may also have negative consequences on social‐emotional development in young children. Consequently, educating and providing parents with strategies on how to establish healthy sleep habits in infancy and early childhood may protect against adverse social‐emotional and behavioral development outcomes.

Children who did not attend an informal playgroup were more likely to show delays in social‐emotional competencies than children who attended informal playgroups. We posit that the impact of attending informal playgroups on child social‐emotional development may be twofold: (1) they provide an opportunity for mothers to receive social support, previously identified as a protective factor for child development outcomes^33^ and (2) they provide an opportunity for child socialization, which is associated with the development of social competence.^34^ Parenting programs and public health professionals could inform parents about the potential benefits of informal playgroups, including opportunities for social support and child socialization, and how these can positively impact child development. Decision makers could encourage communities to provide spaces for parents to congregate to build social support networks, which would enhance well‐being for children and families.

Mothers who reported lower levels of optimism during pregnancy were more likely to have a child with behavioral problems at 2 years of age than mothers with higher levels of optimism during pregnancy. Optimistic individuals adapt to stressors more effectively,^35^ and mothers who are optimistic may more easily adapt to their new role as a parent and the associated challenges of raising a young child. Optimism has been linked to positive parenting practices, including competence‐promoting parenting practices and effective child management.^36^ These findings linking maternal optimism to improved coping with stress and positive parenting practices may help to explain how maternal optimism may play a role in children's behavioral development. Although dispositional optimism is a relatively stable trait, research also indicates that, to some extent, optimism can be taught^37^ and, therefore, parenting intervention and prevention programs for behavior development in young children could include content aimed at cultivating maternal optimism.^36^

Mothers were asked to describe their ability to fulfill family, work, or other responsibilities (eg, volunteer work, household duties, and other children) since giving birth to their child. Mothers who reported “it is difficult most or all of the time” were more likely to have a child with behavior problems at 2 years of age compared with mothers who reported “it is never or sometimes difficult.” This concept of challenges and conflict among women's life roles has been well documented in the literature^38^; however, we did not find any previous studies that directly examined the relationship between balancing family, work, and life roles, and child development. Mothers who experience more challenges balancing the conflicting demands from their family, work, and life are likely experiencing challenges with coping. Poor maternal coping has been found to be associated with poor child functioning, including behavior problems, poorer child self‐regulation, and emotional difficulties.^39^ Challenges with balancing roles is also associated with a number of indicators of maternal wellbeing, including increased rates of depression, stress, anxiety, and lower life satisfaction.^40^ Supporting new parents with strategies to prevent parents from feeling overwhelmed with balancing their roles and responsibilities provides an opportunity to improve maternal wellbeing, and our results extend the current findings suggesting an opportunity to protect against early child behavioral problems as well.

Having at least 1 hour of daily screen time on any type of media (television, DVDs, movies, computer, tablet) was associated with an increased risk of behavioral problems at age two. A longitudinal study has previously reported an association between early childhood television exposure and behavior problems,^41^ although this study only examined television viewing time, not total screen time in preschool aged children. Our analysis adds to the current literature by providing support of an association between all forms of screen time and child behavior problems. As society (including preschool aged children) spends more time on tablets, smartphones, and computers, it will be important to consider screen time from all types of media, not just television viewing. To protect against behavior problems at age two, limiting all forms of screen time to less than 1 hour per day may be an important and fairly simple strategy to share with parents of young children, and aligns with recommendations from the Canadian Pediatric Society.^42^

Children who were exposed to a second language on a regular basis were more likely to have behavior problems at age two than children in single language home settings. The association between exposure to a second language and child behavior has not previously been reported. From the current study, this was a single question that did not ask about the number of hours children were exposed to a second language; therefore, more refined analyses are warranted to investigate this relationship. It is also possible that this variable is a proxy for something else, like cultural influences, which may influence parenting style, discipline strategies, and child behavior.

Although our analyses identified independent risk factors, it is likely that these factors cluster together, and if most or all present, compound the risk of suboptimal social‐emotional competence and behavior; this is suggested by the high predicted probabilities for the outcomes when all risk factors are considered present. Further research could examine a risk profile for suboptimal child outcomes in early childhood, and intervention strategies that target a range of risk factors would be beneficial. For example, many of the factors associated with adverse outcomes in preschool children could be addressed through strategies that normalize help‐seeking behavior for parents of newborns and encourage use of low‐cost community resources. Many parenting programs address content related to efficacy, sleep, mental health, community resources, and child development. Health and child care providers could encourage parents to engage with existing supports and services, which may have the compound benefit of providing parents with skills and strategies for parenting, while creating supportive relationships. Our results also suggest that universal intervention strategies could target maternal depression in early childhood and child sleep hygiene, given that these risk factors were significant for both outcomes. Interaction factors and community engagement were specific to child social‐emotional competencies, while maternal coping, and exposure to screens and a second language were specific to child behavior. Further research is warranted to confirm these factors, which would inform targeted interventions.

### 4.1 Study limitations

Our outcome measure, the BITSEA, which provided measures of children's social‐emotional competencies and behavior problems, was based on maternal report; it is a screening instrument, and, therefore, follow‐up and more comprehensive evaluation is needed to rule in a diagnosis of a developmental delay or behavioral problem. Multi‐informant information would be valuable for identification of at‐risk children, such as teacher reports in addition to parental reports. Similarly, all independent predictor data was obtained through participants completing questionnaires, and, therefore, outcomes such as maternal depression and anxiety were not based on clinical diagnosis. However, the measures selected for use in the AOB/F study are standardized measures that are widely used by researchers with established satisfactory psychometric properties. The BITSEA and other standardized measures were not validated using a Canadian population; however, previous work shows that maternal mental health measures are valid in the AOF cohort,^43^ and we have no reason to expect that the BITSEA would perform differently in this sample as compared with the validation sample.

Our analyses are restricted to identifying risk factors for the occurrence of social‐emotional delays and behavior problems at 2 years of age. We are, therefore, unable to examine the extent to which these factors increase the risk for poor outcomes across time. In addition, given that some single items in the BITSEA replicate risk factor constructs (eg, sleep problems) there may be a slight overestimation of the association between factors present at both the exposure and outcome level, and we acknowledge the potential bi‐directional relationships between factors measured at the same time point (eg, child behavior and each of parental self‐efficacy and work‐life balance). Although a similar bi‐directionality might exist between maternal depression and child outcomes, we also are ensuring that reporting bias is accounted for, given that mothers with depressive symptoms might overestimate problematic behavior in their children. There is also potential for reporting bias among mothers with low optimism who may overestimate behavioral challenges in their children. Finally, there is the potential for selection bias and limits to generalizability, given that participants who completed the 2‐year follow‐up differed from those eligible, in that they were more likely to have higher education and income, be married, and Caucasian. As such, caution is warranted in the interpretation of the results in the context of families that are more vulnerable.

## 5 CONCLUSIONS

Our study extends prior research by analyzing data from a large‐scale, community‐based pregnancy cohort, the AOB/F study, which is representative of the parenting and child population in an urban center in Canada. The AOB/F study also provided the unique opportunity to comprehensively examine an extensive number of contemporary factors, as well as socio‐demographic, gestational, and birth variables. This analysis allowed for the evaluation of numerous factors simultaneously, and the predicted probability of poor outcomes for these exposures. A venue for further research in this cohort would be to examine longitudinal trajectories of children identified at risk, as well as mechanisms underlying associations across time and subgroups of families that would benefit from targeted intervention approaches.

Despite this comprehensive approach to considering a large number of risk factors from the prenatal, birth, early infancy, and early childhood periods, our results showed that in general, the factors most predictive of poor child behavior and social‐emotional development co‐occurred with developmental outcomes at age two. Risk factors co‐occur in families and communities, and the high predicted probability of adverse outcomes associated with exposure to multiple risk factors highlights the need to identify families early and invest appropriately for optimal outcomes.^44^ The importance of a diverse array of environmental factors can help guide strategies that normalize help seeking and uptake of parenting programs and engagement in community social support opportunities.

Identifying and providing support and intervention to mothers of young children experiencing poor mental health is critical to improving women's mental health and children's psychosocial development. Population‐based strategies that support parents of young families in establishing healthy sleep habits, engaging in daily play, attending informal playgroups, and limiting screen time would enhance children's behavior and social‐emotional development at age two.

## CONFLICT OF INTEREST

The authors declare that they have no conflicts of interest.

## AUTHOR CONTRIBUTIONS

Conceptualization: Sheila McDonald, Heather Kehler, Suzanne Tough

Formal Analysis: Heather Kehler

Methodology: Sheila McDonald, Heather Kehler, Suzanne Tough

Writing‐Original Draft Preparation: Sheila McDonald, Heather Kehler

Writing‐Review and Editing: Sheila McDonald, Heather Kehler, Suzanne Tough
